# Wheat ATI CM3, CM16 and 0.28 Allergens Produced in *Pichia Pastoris* Display a Different Eliciting Potential in Food Allergy to Wheat ^‡^

**DOI:** 10.3390/plants7040101

**Published:** 2018-11-16

**Authors:** Silvio Tundo, Roberta Lupi, Mickael Lafond, Thierry Giardina, Colette Larré, Sandra Denery-Papini, Martine Morisset, Raviraj Kalunke, Francesco Sestili, Stefania Masci

**Affiliations:** 1 Department of Agriculture and Forest Science (DAFNE), University of Tuscia, Via S. C. de Lellis snc, 01100 Viterbo, Italy; silvio.tundo@unipd.it (S.T.); rkalunke@gmail.com (R.K.); francescosestili@unitus.it (F.S.); 2 Present address: Department of Land, Environment, Agriculture and Forestry (TESAF), University of Padova, Viale dell’Università 16, 35020 Legnaro (PD), Italy; 3 UR 1268 BIA (Biopolymers, Interactions, Assemblies) INRA, 44300 Nantes, France; roberta.lupi@inra.fr (R.L.); colette.larre@inra.fr (C.L.); sandra.denery@inra.fr (S.D.-P.); 4 Aix Marseille Univ, CNRS, Centrale Marseille, iSm2, Marseille, France; mickael.lafond@univ-amu.fr (M.L.); thierry.giardina@univ-amu.fr (T.G.); 5 Service d’Allergologie - CHU ANGERS, 4 rue Larrey 49933 Angers Cedex 9, France; Martine.Morisset@chu-angers.fr (M.M.); 6 Present address: Institute of Plant and Microbial Biology, Academia Sinica, Taipei, Taiwan

**Keywords:** wheat, alpha-amylase/trypsin inhibitor, heterologous expression, *Pichia pastoris*, allergy

## Abstract

Although wheat is a staple food for most of the human population, some of its components trigger adverse reactions. Among wheat components, the alpha-amylase/trypsin inhibitors (ATI) are important triggers of several allergies and activators of innate immunity. ATI are a group of exogenous protease inhibitors and include several polypeptides. The three ATI polypeptides named CM3, CM16 and 0.28 are considered major allergens, and might also play a role in other common wheat-related pathologies, such as Non Celiac Wheat Sensitivity and even Celiac Disease. On this basis, we pointed to obtain high amounts of them in purity and to evaluate their allergenicity potential. We thus isolated the mRNA corresponding to the three ATI genes CM3, CM16 and 0.28 from 28 days post-anthesis wheat kernels and the corresponding cDNAs were used for heterologous expression in *Pichia pastoris*. The three purified proteins were tested in degranulation assay against human sera of patients with food allergy to wheat. A large range of degranulation values was observed for each protein according to the sera tested. All of the three purified proteins CM3, CM16 and 0.28 were active as allergens because they were able to induce basophils degranulation on wheat allergic patients’ sera, with the highest values of β-hexosaminidase release observed for CM3 protein.

## 1. Introduction

Wheat (*Triticum* spp.) is one of the most important food grain sources for humankind providing starch and about 20% of global dietary protein worldwide, but it can be associated with adverse reactions in predisposed subjects. Such reactions involve specific immunological mechanisms in the case of wheat allergies and celiac disease (CD), a permanent hypersensitivity to wheat gluten proteins with an autoimmune component. Intolerances, such as Non Celiac Wheat Sensitivity (NCWS) also known as Non Celiac Gluten Sensitivity (NCGS), whose mechanism has not been clarified yet, are also described, particularly in a context of Irritable Bowel Syndrome (IBS) [1].

Wheat allergies are classified according to exposition routes into food allergy, respiratory allergy, and contact urticaria [2]. However, food allergy can affect both the gastrointestinal and the respiratory tracts [3]. Moreover, Wheat Dependent Exercise-Induced Anaphylaxis (WDEIA) is a particular wheat allergy triggered by co-factors such as exercise and alcohol. Among respiratory allergies, baker’s asthma affects about 10% of wheat flour workers and is the main cause of occupational respiratory disease in Western countries [4]. Baker’s asthma is well characterized: among the major allergens implicated there are the alpha-amylase/trypsin inhibitors (ATI) [4,5] and the same protein class is also involved in some patients suffering from food allergy to wheat [6,7], notably those exhibiting symptoms of atopic dermatitis, as well as, though to a minor extent, to WDEIA [8]. Antibodies directed to some members of this family have also been detected in sera from patients with CD [9], but their putative pathogenic role is yet unknown. Since few years, wheat ATI were clearly revealed as activators of innate immunity [10]. This activity leads to several hypotheses on their potential role in the digestive symptoms described by NCWS or CD patients [11,12]. Recently, their capacity to exacerbate allergic inflammation was shown in a humanized mouse model [13].

It is a common opinion that adverse reactions to wheat are increasing in recent years but it is a matter of debate if such an increase is true.

In regard to true allergies there is uncertainty, especially because there is a great heterogeneity in estimates. The situation of NCWS is even worse, because of the absence of biological markers that make the diagnosis difficult [14]. Whatever the real situation, there is an increased consumers’ concern towards this aspect, that needs to be taken into consideration.

At this regard, the possibility to detect the amount of specific allergens or, in general, of components triggering adverse reactions in wheat-based food is of fundamental importance for people with wheat-related pathologies.

ATI are extractable with the wheat salt soluble protein fraction that includes albumins and globulins. Three groups of ATI have been described to be active against insects and mammalian alpha-amylases, but not against cereal enzymes (for a review see [15]). These include the 12 kDa monomeric inhibitors, also known as 0.28 proteins, encoded by genes on the short arms of the group 6 chromosomes; the 24 kDa homodimeric inhibitors, also known as the 0.19 and 0.53 proteins, encoded by genes on the short arms of the group 3 chromosomes [16]; the third group is constituted of the 60 kDa heterotetrameric inhibitors. This group includes proteins characterized by specific solubility in chloroform/methanol (CM) mixtures, that accounts for their belonging to the so-called CM protein types (for review see [17]). CM proteins are generally composed of one copy of either CM1 or CM2, encoded by genes on chromosomes 7D or 7B, plus one copy of either CM16 or CM17, encoded by genes on chromosomes 4B or 4D, plus two copies of CM3, also encoded on chromosomes 4B or 4D [18]. 

Isolation of single isoforms of water/salt-soluble wheat allergens is hard to accomplish. Wheat seed proteins, either gluten or non gluten proteins (among these latter there are ATI) belong to the prolamin superfamily [19], thus they show high homology that is at the basis of difficulties in their purification. Moreover they are very abundant in terms of number of polypeptides. Two dimensional protein patterns of seed proteins present in the bread wheat cultivar Bobwhite (from which the three genes here used were isolated) are reported in our previous papers [20,21]. However, protein isolation is essential for specific studies. For example, Ṧotkovskỳ and colleagues [22] set up a three-step isolation protocol, consisting of ultrafiltration, native (liquid-phase) isoelectric focusing and affinity chromatography (HPLC) and highlighted 27 IgE-binding wheat proteins, including the ATI inhibitors 0.19, 0.53, 0.28 and 0.19 dimeric, and the ATI CM1, CM2, CM3, CM16 and CM17. Among the proteins reacting with patient’s IgE, the most frequently IgE recognized ATI were the monomeric 0.28 protein and the subunits CM16 and CM3. In particular CM3 and CM16 isoforms were involved in WDEIA [8]. Kusaba-Nakayama and colleagues [23] compared the capacity of CM3, CM2 and CM16 to cross Caco-2 monolayer cells finding that CM3 is the best form found in basolateral compartment. On the basis of the observation that ATI proteins 0.28, CM16 and CM3 are major allergens, that might also play a role in other common wheat-related pathologies, such as NCWS and CD, and that ATI bioactivity (and likely amount as well) may vary among different wheat genotypes [10], we deemed it useful to obtain high amounts of these specific three ATI proteins in purity, with the aim of evaluating the allergenicity potential of these purified proteins compared to the total CM-like protein fraction extracted from wheat kernels, and eventually producing antibodies that can be used to test the presence and the amount of such ATI proteins in different wheat genotypes and derived products.

In order to obtain pure proteins, heterologous expression in *Pichia pastoris* was the method of choice. The mRNAs, corresponding to the three ATI genes *CM3*, *CM16* and *0.28*, were extracted from 28 days post-anthesis caryopses of *Triticum aestivum* cv Bobwhite and the corresponding cDNAs were used. The three proteins were expressed, purified and tested in degranulation assay using human sera of patients with food allergy to wheat.

## 2. Results

### 2.1. Cloning of CM3, CM16, 0.28, Expression in P. Pastoris and Purification

**Figure 1:**
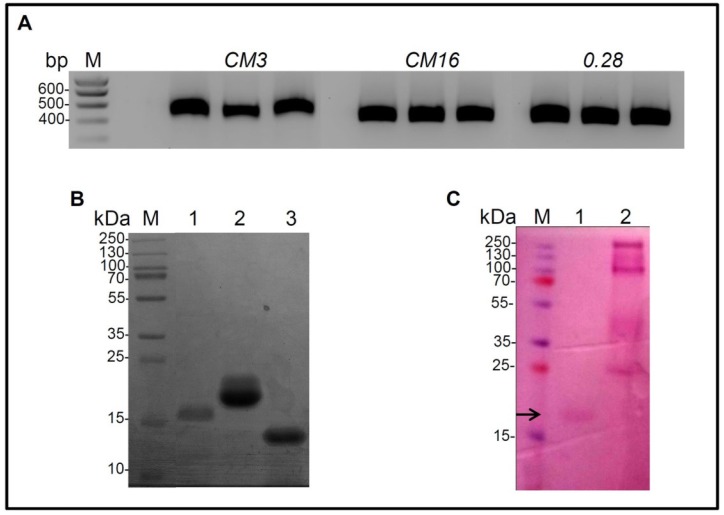
Production of recombinant CM3, CM16 and 0.28 allergens. (**A**) Agarose gel of the Reverse Transcription-PCR (RT-PCR) performed on cDNA of 28 days post anthesis *Triticum aestivum* cv. Bobwhite kernels by using specific primers for alpha-amylase/trypsin inhibitors (ATI) genes *CM3*, *CM16* and *0.28*. The three bands per sample correspond to technical replicates. M: marker. (**B**) Sodium dodecyl sulfate polyacrylamide gel electrophoresis (SDS-PAGE) analysis of purified ATI proteins heterologously expressed in *Pichia pastoris* after Ni-Nta purification and concentration steps. Samples: (M) Molecular mass marker; (1) Purified CM3; (2) Purified CM16; (3) Purified 0.28. (**C**) SDS-PAGE gel of purified CM16 protein subjected to periodic acid Schiff reagent staining for protein glycosylation detection. Samples: M) Molecular mass marker; (1) purified CM16 protein and (2) Positive control. The arrow indicates the band corresponding to CM16.

Amplicons corresponding to the three cDNA clones are reported in Figure 1A. 

The sizes of the three amplicons correspond to the expected ones calculated on the basis of the nucleotide sequence (507 bp for CM3, 432 bp for CM16 and 456 bp for 0.28).

As regards the 0.28 gene, we followed indications coming from previous papers (among which Sanchez-Monge and colleagues [17]) reporting that the mature protein begins at position 31 (thus with SGPW-), differently from what indicated by the SignalP software used for prediction.

A first attempt to separately express CM3, CM16 and 0.28 proteins was done in Escherichia coli Rosetta Gami strain. The coding regions of the three genes were cloned in the pOPINE vector and the transformed E. coli cells were induced with isopropyl B-D-thiogalactoside (IPTG) but the proteins were not expressed either in the soluble or insoluble fraction (data not shown). This result prompted us to use the eukaryotic *P. pastoris* expression system, thus the following results are relative to this latter expression system.

**Figure 2:**
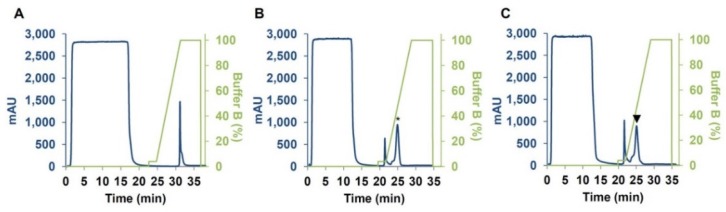
Chromatograms of CM3 (**A**), CM16 (**B**) and 0.28 (**C**) using HisTrap HP affinity column. Chromatograms indicate the Absorbance Units (at 280 nm) at each retention time (min) and following the elution buffer concentration. The peaks corresponding to CM16 and 0.28 proteins are indicated by an asterisk and a triangle, respectively. Buffer B is 50 mM Na-phosphate, 150 mM NaCl, 500 mM Imidazole, pH 7.5.

Large-scale expression was carried out as previously described by Lafond and colleagues [24] with slight modifications in Pichia pastoris expression system. After the induction step with methanol, P. pastoris supernatants were subjected to an ultrafiltration step followed by subsequent one-step purification on immobilized metal-ion affinity column (i.e., HisTrap HP 5 mL, Figure 2) yielding 0.080, 0.45 and 0.32 mg/mL^−1^ of CM3, CM16 and 0.28, respectively.

These enzyme preparations were found to be >90% pure by sodium dodecyl sulfate polyacrylamide gel electrophoresis (SDS–PAGE) analysis, estimated as pure to homogeneity and considered for the rest of the study. A single major protein band for each of the expressed proteins CM3, CM16 and 028 was present with an apparent molecular weight of 16 kDa, 20 kDa and 14 kDa respectively after SDS-PAGE analysis (Figure 1B).

The molecular weight on electrophoresis gel of CM3 and 0.28 corresponded to the expected ones, whereas the CM16 observed molecular weight was much higher than the theoretical one (i.e. 14 kDa) (Figure 1B), suggesting that the recombinant CM16 could be glycosylated (Figure 1C).

**Figure 3:**
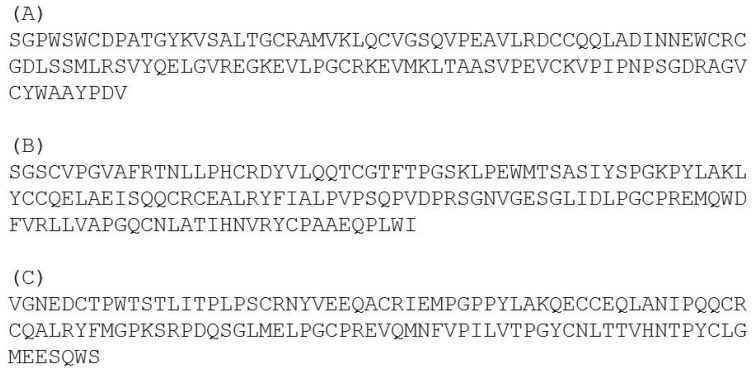
Identification of the three ATI polypeptides by LC-MS/MS. (**A**) 0.28; (**B**) CM3 and (**C**) CM16. Matching peptide sequences are underlined.

The observation of recombinant protein glycosylation by the yeast host is consistent with the in silico analysis by the NetNGlyc server, which predicted one glycosylation site in CM16 at position N124. In contrast, no N-glycosylation site was predicted in CM3 and 0.28. The presence of sugars linked to the protein was established by periodic acid Schiff staining, and those reported by Sanchez-Monge and colleagues [25] relatively to the same subunit present in the durum wheat cultivar Senatore Cappelli, confirming the result of the in silico analysis (Figure 1C). Protein identities were confirmed after tryptic digestion followed by a LC-MS⁄MS analysis, as accurately described in the Material and Method section, and showed 62, 79 and 100% of peptide coverage for CM16, CM3 and 0.28, respectively. Protein identifications are reported in Figure 3.

The observation of recombinant protein glycosylation by the yeast host is consistent with the in silico analysis by the NetNGlyc server, which predicted one glycosylation site in CM16 at position N124. In contrast, no N-glycosylation site was predicted in CM3 and 0.28. The presence of sugars linked to the protein was established by periodic acid Schiff staining, and those reported by Sanchez-Monge and colleagues [25] relatively to the same subunit present in the durum wheat cultivar Senatore Cappelli, confirming the result of the in silico analysis (Figure 1C). Protein identities were confirmed after tryptic digestion followed by a LC-MS⁄MS analysis, as accurately described in the Material and Method section, and showed 62, 79 and 100% of peptide coverage for CM16, CM3 and 0.28, respectively. Protein identifications are reported in Figure 3.

### 2.2. Capability of CM3, CM16 and 0.28 to Induce RBL-SX38 Cell Activation Compared to CM-Like Protein Extract

**Figure 4:**
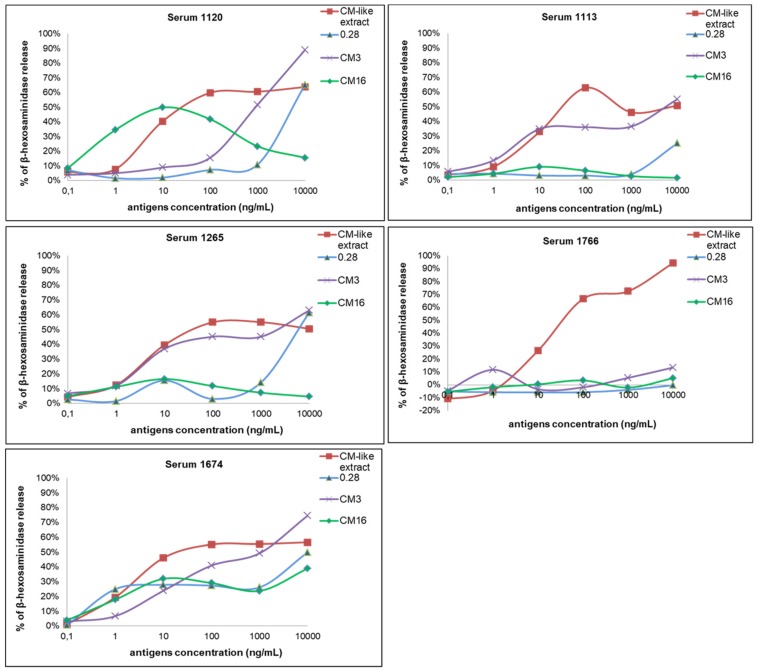
Basophil activation assay with five sera from wheat allergic patients. RBL-SX38 cells were stimulated with concentrations ranging from 0.1 to 10.000 ng/mL of CM3, 0.28, CM16 proteins and of CM-like extract from Bobwhite.

The sera selected in this work displayed high concentrations in specific IgE directed to the albumin/globulin fraction and/or to the CM-like protein fraction (available for 13 sera). The triggering capacity of the three CM-like proteins and of the total CM-like proteins extract were analyzed at different protein concentrations using five sera from wheat allergic patients (Figure 4).

**Figure 5:**
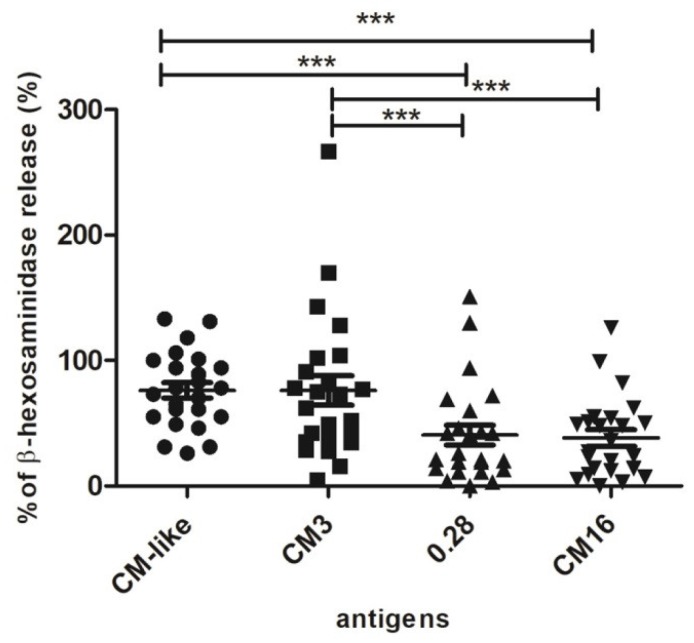
Basophil activation assay with 23 sera from wheat allergic patients. RBL-SX38 cells were stimulated with 1 µg/mL of CM3, 0.28, CM16 proteins and with CM-like extract from Bobwhite. *** *p* < 0.0001.

The degranulation curves observed with the CM-like extract were similar with the five sera, and reached a maximum of β-hexosaminidase release and a plateau at 100 ng/mL. On the opposite, the values and protein concentrations of the maximal percentage of β-hexosaminidase release differed between the CM-like proteins as a function of sera. The protein concentration of 1 µg/mL inducing a response within the plateau for the reference CM-like extract was selected to compare the eliciting capacity of the three CM-like proteins in presence of all the sera. The three CM-like proteins and the extract did not induce any cell activation in presence of the control sera (data not shown). On the contrary, the CM-like protein fraction triggered activation of basophils when cells were sensitized with the 23 sera from wheat allergic patients. A large range of degranulation values was observed for each of the CM-like proteins according to the sera tested. High values of β-hexosaminidase release were observed for the total CM-like protein extract and for pure CM3, with relative percentages of degranulation ranging from 26% to 133% and from 28% to 267%, respectively. These two fractions induced more than 50% of RBL degranulation for 86% and 65% among the 23 sera tested, whereas 0.28 and CM16 induced more than 50% of degranulation with only 30% and 37% of the sera. Actually, the comparison of percentage of β-hexosaminidase release showed a statistically significant difference between the total CM-like protein fraction from Bobwhite and CM16 and 0.28. On the contrary, CM3 showed the same degranulation capacity of the positive control. Consequently, statistically significant differences were also observed between CM3 and CM16 as well as between CM3 and 0.28 (Figure 5).

## 3. Discussion

Whether the increase of adverse reactions to wheat are a matter of fact or not, is still under discussion. The increase of CD might be real [26,27], either because of the supposed increased exposure to gluten [28], or because of the increased sensitivity of diagnosis, or as a consequence of natural selection. Although CD undoubtedly has multifactorial aspects, it is not possible to note that, up to a few decades ago, non-diagnosed CD people could not reach reproductive age; moreover CD women may show difficulties in reproduction. The present possibility to make early diagnoses of CD allows CD people following a Gluten Free diet to have a normal life, including reproduction, and this might increase the frequency of the genetic predisposition to CD.

In regard to true allergies there is uncertainty, especially because there is a great heterogeneity in estimates [29]. The situation of NCWS is even worse, because of the absence of biological markers that make the diagnosis difficult [14]. Whatever the real situation, there is an increased perception by consumers towards this aspect, that needs to be taken into consideration. In this regard, the possibility to detect the amount of specific allergens or, in general, of components triggering adverse reactions in wheat-based food is of fundamental importance for people with wheat-related pathologies. Moreover, as stated in a recent paper [30], diagnoses of several wheat allergies, specifically the occupational ones, are difficult because of great variability among individuals; but the possibility to have available single wheat components might help in distinguish between sensitization, allergy and wheat seropositivity due to cross-reactivity to grass pollen.

In this context, we have heterologously expressed three ATI proteins, CM3, CM16 and 0.28, that are considered major allergens and are also involved in WDEIA [8] and might play a role as well in NCWS and CD [11,12]. This system in fact allows producing high amount of pure proteins, which are very difficult to obtain by in vivo extraction. The three purified proteins were tested in degranulation assay in presence of human sera from patients with food allergy to wheat. All the three purified proteins CM3, CM16 and 0.28 were active as allergens because they were able to induce a degranulation of the basophils sensitized with the IgE from these wheat allergic patients. However, a large range of degranulation values was observed for each protein according to the sera tested. Important was the observation that the highest values of β-hexosaminidase release were observed for CM3 protein, whose values were comparable of those observed in the positive control, corresponding to the whole CM protein fraction, accounting for the important role played by this polypeptide in triggering allergic reaction. This result is in contrast with Sanchez-Monge and colleagues [25] for whom the glycosylated form of CM16 showed the strongest IgE-binding capacity in immunoblots, at least for patients with baker’s asthma. This clearly shows the importance to use complementary technique and functional assays to test the allergenic activity of proteins.

Moreover, it is noteworthy the fact that recent works indicate that CM3 has a main function in initiating the innate immune response [11,31].

On the basis of these results, the three proteins will be used to produce polyclonal and monoclonal antibodies with the aim of testing the presence and the amount of such ATI proteins in different wheat-based food matrices. Moreover, such antibodies can be used to evaluate the presence of residual ATI proteins in properly developed wheat genotypes in which ATI genes have been silenced either by RNA interference or genome editing technologies (our group, in preparation).

## 4. Materials and Methods 

### 4.1. Plant Material and Genes Isolation

Total RNA was extracted from 28 days post-anthesis kernels of the bread wheat cultivar Bobwhite using the RNeasy Plant Mini Kit (Qiagen Italia, Milan, Italy) according to manufacturer’s instructions and first strand cDNA was obtained using the QuantyTect® Reverse Transcription kit (Qiagen Italia, Milan, Italy). Primers were designed using the GenBank accession numbers AY436554.1, X17573.1 and AJ223492.1, respectively relative to CM3, CM16 and 0.28.

cDNAs were amplified by Reverse Transcription-PCR (RT-PCR) using the primers CM3_F/CM3_R for CM3, CM16_F/CM16_R for CM16, 0.28_F/0.28_R for 0.28 (sequences are listed in Table S1). High fidelity AccuPrime Taq DNA Polymerase was from ThermoFisher Scientific (Waltham, MA, USA). The PCR was carried out by repeating for 25 times the following cycle: 1 min at 94 °C; 45 s at 60 °C; 1 min at 68 °C. Amplification products were purified by using the Wizard® SV Gel and PCR Clean-Up System (Promega Italia s.r.l. Milan, Italy), cloned into pGEM-T Easy Vector (Promega Italia s.r.l. Milan, Italy) and checked by nucleotide sequencing. Sequence analyses were performed using the DNAMAN software (Lynnon Biosoft, Quebec, CA) that uses the ClustalW algorithm for multiple sequence alignment. The region encoding the mature protein of each gene was inserted into the pPICZαA vector using the In-FusionTM Advantage PCR Cloning kit (Clontech Laboratories, Mountain View, CA, USA) with primers CM3_For_inf/CM3_Rev_inf, CM16_For_inf/ CM16_Rev_inf and 0.28_For_inf/0.28_Rev_inf (sequences are listed in Table S1). The PCR was carried out by repeating for 30 times the following cycle: 10 s at 98 °C; 30 s at 60 °C; 1 min at 72 °C. The three cDNAs were eventually cloned in pOPINE vector (Addgene, Cambridge, UK) that allows the production of recombinant proteins as fusion to an N-terminal polyhistidine tag (6 His).

The cDNAs coding for the CM3, CM16, and 0.28 were fused-in-frame to the nucleotide sequence of the *Saccharomyces cerevisiae* α-factor secretion signal under the control of the AOX1 promoter of p*PICZαA* expression vector, to finally obtain the p*PICZαA_CM3*, p*PICZαA_CM16* and p*PICZαA_028* constructs. A six-histidine tag was added at the N-terminal end of the target proteins. The resulting recombinant expression plasmids were linearized with PmeI for integration into *P. pastoris* genome. 

### 4.2. Expression and Purification of CM3, CM16 and 0.28 

The p*PICZαA* expression vector and the expression kit used, including the *P. pastoris* strain X-33, zeocin, and oligonucleotides, were from Invitrogen (Groningen, Netherlands).

The linearized DNAs were introduced for electroporation into *P. pastoris* strain X-33 using a Multiporator® (Eppendorf, Hamburg, Germany) at 1500 V for 5 ms. The transformants were selected using Minimal Methanol and Minimal Dextrose plates. Finally, in order to select multicopy vector strain transformants, YPDS (Yeast Peptone Dextrose Sorbitol) plates containing 100 and 500 μg/mL zeocin® were used. Large-scale expression was carried out as previously described by Lafond and colleagues [24] with a slight protocol modification. For each construct, the most producing clone was grown for 16 h in 1 L Buffered Glycerol-complex Medium (BMGY) in 3 L baffled flask and then the cells were transferred to 200 mL Buffered Methanol-complex Medium (BMMY) in 1 L baffled flask at 200 rpm and 30 °C. The culture supernatant was recovered after 3-days with 3% (*v/v*) methanol induction by centrifugation (10 min, 4000× *g*) and the culture supernatant was concentrated using UltracelTM ultrafiltration membrane and nitrogen atmosphere (3 kDa cut-off, Poly-Ether-Sulfone, 4 bar, Millipore, Molsheim, France) in order to have a lower final volume. This latter was subjected to one purification step on a 5 mL HisTrap HP column (GE Healthcare, Piscataway, NJ, USA) connected to FPLC® equipment (Akta Purifier 10, GE Healthcare, Piscataway, USA) equilibrated with 50 mM Na-phosphate buffer, pH 7.5, 150 mM NaCl and eluted with 50 mM Na-phosphate buffer, pH 7.5, 150 mM NaCl, containing 500 mM Imidazole at a flow rate of 2 mL/min.

### 4.3. In Silico Prediction of N-Glycosylation

*In silico* analysis for signal peptide prediction was performed to identify the three mature proteins encoded by their corresponding genes. The deduced proteins CM3 and CM16 contain a signal peptide of 25 and 24 residues, respectively. 

The possible presence of N- or O-glycosylation sites in CM3, CM16, and 0.28 sequences was predicted using NetNGlyc 1.0 server (http://www.cbs.dtu.dk/services/).

### 4.4. Polyacrylamide Gel Electrophoresis and Glycosylation Staining 

SDS-PAGE analysis was performed in 15% (*w/v*) polyacrylamide gel as described by Laemmli and colleagues [32] under reducing conditions. Proteins were visualized by Instant blue (Expedeon, Swavesey, UK) staining. For glycosylation determination, gels were stained for carbohydrate detection with periodic acid-Schiff (PAS) using a SIGMA-ALDRICH glycoprotein staining kit® according to the manufacturer’s protocol. The protein concentration was determined using the Bio-Rad protein assay kit with bovine serum albumin as standard. 

### 4.5. Protein Identification by Mass Spectrometry

Proteins were in-gel digested by trypsin (Promega). Resulting peptides were analyzed by LC-MS/MS using an ESI-Q-Exactive Plus mass spectrometer (ThermoFisher Scientific, San Jose, CA, USA) coupled to an Ultimate 3000 RSLC nano liquid chromatography system (Dionex, Sunnyvale, CA, USA). Peptides were eluted onto a C18 column (Acclaim PepMap RSLC, 75 µm i.d. × 150 mm, 2 µm, 100 Ǻ, Dionex) with a 52 min linear gradient from 6% to 40% of mobile phase B (80% acetonitrile and 0.1% formic acid in water) in A (0.1% formic acid in water). Peptides were detected in a positive ion mode using Top 10 data dependent workflow (extended protocol in Nguyen et al. [33]). Spectra were analyzed with Proteome Discoverer software (ThermoFisher, version 2.1.0.81) using the following parameters: (1) protein database composed of sequences of CM3, CM16 and 028 proteins, human keratins, pig trypsin and *Komagataella pastoris* (NCBI Taxonomy ID 4922, 5317 entries); (2) enzyme used: trypsin (2 missed cleavages); (3) fixed modification: carbamidomethyl (Cys); (4) variable modification: oxidation (Met); (5) precursor mass tolerance: ± 10 ppm; (6) fragment mass tolerance: ± 0.02 Da.

### 4.6. Extraction of Salt Soluble Proteins: CM-Like and Albumin/Globulin Fractions

CM-like protein fraction from the bread wheat cultivar Bobwhite was extracted from milled wheat seeds as described by Hurkman and Tanaka [34]. Briefly, 1 g of flour was suspended in 3 mL of a buffer containing 50 mM Tris-HCl, 100 mM KCl, 5 mM EDTA, pH 7.8 and shaked for 1 h at 4 °C. After centrifugation at 8000 × *g* for 40 min, the supernatant was recovered, mixed with 2.5 volumes of 0.1 M ammonium acetate and stored at −20 °C overnight. Afterwards, the supernatant was separated from the metabolic fraction by centrifugation at 8000 × *g* for 40 min and precipitated with acetone at −20 °C overnight. Pellet was eventually washed twice with acetone and dried. The albumin/globulin (A/G) fraction was extracted from the bread wheat cultivar Récital, and the protein concentration of the two fractions was determined by the BC Assay Protein Quantification Kit (Uptima), as described in Larré and colleagues [35].

### 4.7. Sera from Wheat Allergic Patients 

Sera were obtained from 23 patients with clinically documented food allergy to wheat (Table S2). These sera were selected based on their concentrations in specific IgE for albumins/globulins and/or CM-like protein fraction (when available) measured by F-ELISA (Table 1) as described in Lupi and colleagues [7]. These sera were obtained from the Biological Resource Center (BB-0033-00038) of Clinical Immunology and Allergy Service of Angers University Hospital (France) with the informed consent of the patients. Control sera were obtained from six atopic individuals allergic to grass pollens, without food allergy to wheat.

### 4.8. RBL-SX38 Cell Degranulation Test 

CM3, CM16, and 0.28 proteins were evaluated for their capacity to trigger basophil degranulation and compared to the activity of the CM-like protein extract, using an RBL-SX38 in vitro model, as described by Lupi and colleagues [36]. RBL-SX38 cells expressing human FceRI, were kindly provided by Prof. J.P. Kinet (Harvard Medical School, MA, USA). Cells were sensitized with the sera from five patients and stimulated with concentrations varying from 0.1 to 10,000 ng/mL of each antigen solubilized in PBS. In a second step, the comparison of the CM-like protein activity was performed with cells sensitized with the 23 sera and stimulated at the concentration of 1 µg/mL of antigens. Sera were previously detoxified as described in Claude and colleagues [37] and then diluted from 1:50 to 1:100; non-wheat allergic control sera (IgE against grass pollen) were diluted 1:20. The cells were stimulated with a monoclonal anti-human IgE antibody (clone Le27-NBS01 mouse anti-human IgE-Fc Region Antibody; 500 ng/mL, NBS-C Bioscience, Vienne, Austria) as references. The results are expressed as the percentage of the β-hexosaminidase release induced by the samples compared to the release observed with anti-human IgE antibodies as described in Lupi and colleagues [36].

### 4.9. Statistical Analysis

Statistical analyses were performed using GraphPad Prism 5.02 for Windows software (La Jolla, CA, USA). Data were represented as the mean ± standard error. They were analyzed by one-way ANOVA followed by Bonferroni’s multiple comparison test. Differences were considered significant when p values were below 0.05.

## Figures and Tables

**Table 1:** IgE reactivity of patient sera against albumins/globulins fraction (or total CM-like protein fraction when available) from bread wheat cv Récital.

**IgE Concentration (ng/mL)**			
**Patient #**	**Total IgE**	**Specific to A/G Fraction**	**Specific to CM-like Protein Fraction**
**1674**	1468	208	250
**1265**	11486	>500	nd
**1113**	6180	141	nd
**1120**	616	100	nd
**1766**	423	123	120
**134**	2000	196	nd
**610**	sat	281	nd
**639**	8150	333	nd
**642**	524	154	274
**779**	815	110	nd
**1026**	2735	164	nd
**1157**	11113	119	nd
**1266**	13098	140	nd
**1353**	363	1	*243*
**1494**	1454	327	386
**1572**	11142	253	368
**1638**	6741	300	360
**1747**	2218	192	248
**1826**	35024	432	>500
**1829**	14661	123	290
**1830**	1854	137	171
**1862**	4724	216	221
**1875**	5609	>500	>500
**Atopic Serum (Allergic to Grasses Pollen)**			
**#**	**Total IgE**		
**319**	2800		
**322**	3123		
**997**	1471		
**1240**	1253		
**1246**	1112		
**1617**	940		

## Supplementary Materials

The following are available online at http://www.mdpi.com/2223-7747/7/4/101/s1. Table S1 List of primers used for cloning CM3, CM16 and 0.28 genes, Table S2. Serum identification and clinical characteristics of patients allergic to wheat.

## References

[1] Reese I., Schäfer C., Kleine-Tebbe J., Ahrens B., Bachmann O., Ballmer-Weber B., Beyer K., Bischoff S.C., Blümchen K., Dölle S. (2018). Non-celiac gluten/wheat sensitivity (NCGS)—A currently undefined disorder without validated diagnostic criteria and of unknown prevalence. Allergo J. Int..

[2] Sapone A., Bai J.C., Ciacci C., Dolinsek J., Green P.H.R., Hadjivassiliou M., Kaukinen K., Rostami K., Sanders D.S., Schumann M. (2012). Spectrum of gluten-related disorders: Consensus on new nomenclature and classification. BMC Med..

[3] Elli L., Branchi F., Tomba C., Villalta D., Norsa L., Ferretti F., Roncoroni L., Bardella M.T. (2015). Diagnosis of gluten related disorders: Celiac disease, wheat allergy and non-celiac gluten sensitivity. World J. Gastroenterol..

[4] Salcedo G., Quirce S., Diaz-Perales A. (2011). Wheat allergens associated with Baker’s asthma. J. Investig. Allergol. Clin. Immunol..

[5] Pastorello E.A., Farioli L., Conti A., Pravettoni V., Bonomi S., Iametti S., Fortunato D., Scibilia J., Bindsley-Jensen C., Ballmer-Weber B. (2007). Wheat IgE-mediated food allergy in European patients: α-amylase inhibitors, lipid transfer proteins and low-molecular-weight glutenins - Allergenic molecules recognized by double-blind, placebo-controlled food challenge. Int. Arch. Allergy Immunol..

[6] Lupi R., Denery-Papini S., Rogniaux H., Lafiandra D., Rizzi C., De Carli M., Moneret-Vautrin D.A., Masci S., Larré C. (2013). How much does transgenesis affect wheat allergenicity? Assessment in two GM lines over-expressing endogenous genes. J. Proteomics.

[7] Lupi R., Masci S., Rogniaux H., Tranquet O., Brossard C., Lafiandra D., Moneret-Vautrin D.A., Denery-Papini S., Larré C. (2014). Assessment of the allergenicity of soluble fractions from GM and commercial genotypes of wheats. J. Cereal Sci..

[8] Zapatero L., Martínez M.I., Alonso E., Salcedo G., Sánchez-Monge R., Barber D., Lombardero M. (2003). Oral wheat flour anaphylaxis related to wheat α-amylase inhibitor subunits CM3 and CM16. Allergy.

[9] Huebener S., Tanaka C.K., Uhde M., Zone J.J., Vensel W.H., Kasarda D.D., Beams L., Briani C., Green P.H., Altenbach S.B., Alaedini A. (2015). Specific nongluten proteins of wheat are novel target antigens in celiac disease humoral response. J. Proteome Res..

[10] Zevallos V.F., Raker V., Tenzer S., Jimenez-Calvente C., Ashfaq-Khan M., Rüssel N., Pickert G., Schild H., Steinbrink K., Schuppan D. (2017). Nutritional Wheat Amylase-Trypsin Inhibitors Promote Intestinal Inflammation via Activation of Myeloid Cells. Gastroenterology.

[11] Junker Y., Zeissig S., Kim S.J., Barisani D., Wieser H., Leffler D.A., Zevallos V., Libermann T.A., Dillon S., Freitag T.L. (2012). Wheat amylase trypsin inhibitors drive intestinal inflammation via activation of toll-like receptor 4. J. Exp. Med..

[12] Catassi C., Elli L., Bonaz B., Bouma G., Carroccio A., Castillejo G., Cellier C., Cristofori F., de Magistris L., Dolinsek J. (2015). Diagnosis of non-celiac gluten sensitivity (NCGS): The Salerno Experts’ Criteria. Nutrients.

[13] Bellinghausen I., Weigmann B., Zevallos V., Maxeiner J., Reißig S., Waisman A., Schuppan D., Saloga J. (2018). Wheat amylase-trypsin inhibitors exacerbate intestinal and airway allergic immune responses in humanized mice. J. Allergy Clin. Immunol..

[14] Catassi C., Alaedini A., Bojarski C., Bonaz B., Bouma G., Carroccio A., Castillejo G., De Magistris L., Dieterich W., Di Liberto D. (2017). The Overlapping Area of Non-Celiac Gluten Sensitivity (NCGS) and Wheat-Sensitive Irritable Bowel Syndrome (IBS): An Update. Nutrients.

[15] Garcia-Olmedo F., Salcedo G., Sanchez-Monge R., Gómez L., Royo J., Carbonero P., Miflin B. (1987). Plant proteinaceous, inhibitors of proteinases and 3-amylases. Oxford Surveys of Plant Molecular and Cell Biology.

[16] Carbonero P., Garcia-Olmedo F., Shewry P.R., Casey R. (1999). A multigene family of trypsin/α-amylase inhibitors from cereals. Seed Proteins.

[17] Sanchez-Monge R., Gomez L., Garcia-Olmedo F., Salcedo G. (1989). New dimeric inhibitor of heterologous α-amylases encoded by a duplicated gene in the short arm of chromosome 3B of wheat (*Triticum aestivum* L.). Eur. J. Biochem..

[18] Altenbach S.B., Vensel W.H., Dupont F.M. (2011). The spectrum of low molecular weight alpha-amylase/protease inhibitor genes expressed in the US bread wheat cultivar Butte 86. BMC Res. Notes.

[19] Kreis M., Forde B.G., Rahman S., Miflin B.J., Shewry P.R. (1985). Molecular evolution of the seed storage proteins of barley, rye and wheat. J. Mol. Biol..

[20] Scossa F., Laudencia-Chingcuanco D., Anderson O.D., Vensel W.H., Lafiandra D., D’Ovidio R., Masci S. (2008). Comparative proteomic and transcriptional profiling of a bread wheat cultivar and its derived transgenic line over-expressing a low molecular weight glutenin subunit gene in the endosperm. Proteomics.

[21] Lupi R., Denery-Papini S., Rogniaux H., Lafiandra D., Rizzi C., De Carli M., Moneret-Vautrin A.D., Masci S., Larré C. (2013). How much does transgenesis affect wheat allergenicity? Assessment in two GM lines over-expressing endogenous genes. J. Proteomics.

[22] Šotkovský P., Sklenář J., Halada P., Cinová J., Šetinová I., Kainarová A., Goliáš J., Pavlásková K., Honzová S., Tučková L. (2011). A new approach to the isolation and characterization of wheat flour allergens. Clin. Exp. Allergy.

[23] Kusaba-Nakayama M., Ki M., Kawada E., Sato M., Ikeda I., Mochizuki T., Imaizumi K. (2001). Intestinal absorbability of wheat allergens, subunits of a wheat alpha-amylase inhibitor, expressed by bacteria. Biosci. Biotechnol. Biochem..

[24] Lafond M., Navarro D., Haon M., Couturier M., Berrin J.G. (2012). Characterization of a broad-specificity β-glucanase acting on β-(1, 3)-, β-(1,4)-, and β-(1,6)-glucans that defines a new glycoside hydrolase family. Appl. Environ. Microbiol..

[25] Sanchez-Monge R., Gomez L., Barber D., Lopez-Otin C., Armentia A., Salcedo G. (1992). Wheat and barley allergens associated with baker’s asthma. Biochem. J..

[26] Rubio-Tapia A., Kyle R.A., Kaplan E.L., Johnson D.R., Page W., Erdtmann F., Brantner T.L., Kim W.R., Phelps T.K., Lahr B.D. (2009). Increased prevalence and mortality in undiagnosed celiac disease. Gastroenterology.

[27] Catassi C., Kryszak D., Bhatti B., Sturgeon C., Helzlsouer K., Clipp S.L., Gelfond D., Puppa E., Sferruzza A., Fasano A. (2010). Natural history of celiac disease autoimmunity in a USA cohort followed since 1974. Ann. Med..

[28] Kasarda D.D. (2013). Can an increase in celiac disease be attributed to an increase in the gluten content of wheat as a consequence of wheat breeding?. J. Agric. Food Chem..

[29] Zuidmeer L., Goldhahn K., Rona R.J., Gislason D., Madsen C., Summers C., Sodergren E., Dahlstrom J., Lindner T., Sigurdardottir S.T. (2008). The prevalence of plant food allergies: A systematic review. J. Allergy Clin. Immunol..

[30] Raulf M. (2018). Allergen component analysis as a tool in the diagnosis and management of occupational allergy. Mol. Immunol..

[31] Cuccioloni M., Mozzicafreddo M., Bonfili L., Cecarini V., Giangrossi M., Falconi M., Saitoh S.I., Eleuteri A.M., Angeletti M. (2017). Interfering with the high-affinity interaction between wheat amylase trypsin inhibitor CM3 and toll-like receptor 4: In silico and biosensor based studies. Sci. Rep..

[32] Laemmli U.K. (1970). Cleavage of structural proteins during the assembly of the head of bacteriophage T4. Nature.

[33] Nguyen P.C., Delorme V., Bénarouche A., Martin B.P., Paudel R., Gnawali G.R., Madani A., Puppo R., Landry V., Kremer L. (2017). Cyclipostins and Cyclophostin analogs as promising compounds in the fight against tuberculosis. Sci Rep..

[34] Hurkman W.J., Tanaka C.K. (2007). Extraction of wheat endosperm proteins for proteome analysis. J. Chromatogr. B. Analyt. Technol. Biomed. Life Sci..

[35] Larré C., Lupi R., Gombaud G., Brossard C., Branlard G., Moneret-Vautrin D.A., Rogniaux H., Denery-Papini S. (2011). Assessment of allergenicity of diploid and hexaploid wheat genotypes: Identification of allergens in the albumin/globulin fraction. J. Proteomics.

[36] Lupi R., Denery-Papini S., Claude M., Tranquet O., Drouet M., Masci S., Larré C. (2018). Thermal treatment reduces gliadin recognition by IgE, but a subsequent digestion and epithelial crossing permits recovery. Food Res. Intern..

[37] Claude M., Lupi R., Bouchaud G., Bodinier M., Brossard C., Denery-Papini S. (2016). The thermal aggregation of ovalbumin as large particles decreases its allergenicity for egg allergic patients and in a murine model. Food Chem..

