# Supplementary material for: Wheat ATI CM3, CM16 and 0.28 Allergens Produced in Pichia Pastoris Display a Different Eliciting Potential in Food Allergy to Wheat ‡
Source: Plants (Basel). 2018 Nov 16;7(4):101. doi: 10.3390/plants7040101 (PMC6313882; doi:10.3390/plants7040101)
Supplement: Supplementary file 1 [file plants-07-00101-s001.zip › Table S1.docx]

**Table S.1** List of primers used for cloning *CM3*, *CM16* and *0.28* genes

| **Primer name** | **Sequence 5´-3´** |
| --- | --- |
| CM3_F | ATGGCGTGCAAGTCCAGCTG |
| CM3_R | TCTCTAGATCCACAGAGGCTG-3 |
| CM16_F | ATGGCGTCCAAGTCCAACTG |
| CM16_R | CTAGCTCCACTGAGACTCCT |
| 0.28_F | ATGTGGATGAAGACCGTGTTCTG |
| 0.28_R | TTGACTAGACGTCCGGATAC |
| CM3_For_inf | GAGAAAAGAGAGGCTGAAGCTTCCGGCAGCTGCGTCC |
| CM3_Rev_inf | TCAATGATGATGATGATGATGGATCCACAGAGGCTGTTCCG |
| CM16_For_inf | GAGAAAAGAGAGGCTGAAGCTGTCGGCAATGAAGATTGCAC |
| CM16_Rev_inf | TCAATGATGATGATGATGATGGCTCCACTGAGACTCCTCCA |
| 0.28_For_inf | GAGAAAAGAGAGGCTGAAGCTGTCGAGTATGGTGCAAGGAGC |
| 0.28_Rev_inf | TCAATGATGATGATGATGATGGACGTCCGGATACGCGG |
