# Supplementary material for: Wheat ATI CM3, CM16 and 0.28 Allergens Produced in Pichia Pastoris Display a Different Eliciting Potential in Food Allergy to Wheat ‡
Source: Plants (Basel). 2018 Nov 16;7(4):101. doi: 10.3390/plants7040101 (PMC6313882; doi:10.3390/plants7040101)
Supplement: Supplementary file 1 [file plants-07-00101-s001.zip › Table S2.docx]

**Table S.2**. Serum identification and clinical characteristics of patients allergic to wheat. Sera from 23 patients with food allergy to wheat were used. Patients’ reactivity was tested by Skin Prick Test and CAP^®^ Phadia. AEDS: atopic eczema dermatitis syndrome; AS: anaphylactic shock; Asth: Asthma; U: urticaria; GI: gastrointestinal symptoms; Results of food /respiratory challenges: pos: positive; L.T.: labial test; nd: not determined.

| **Sera** | **Sex** | **Age**  **(year)** | **symptoms** | **Skin Prick Test (mm) Wheat** | **Skin Prick Test (mm) Gluten** | **Specific IgE CAP® KUI/L Wheat** | **Specific IgE CAP® KUI/L Gluten** | **Food challenge** |
| --- | --- | --- | --- | --- | --- | --- | --- | --- |
| **1674** |  | 11 |  |  |  |  |  | nd |
| **1265** | M | 4 | AEDS | 5 | 6 | 100 | 100 | nd |
| **1113** | M | 11 | AS | 8 | 7 | 42.5 | 43.5 | Pos 1 g |
| **1120** | M | **2** | URT | 3 | 4 |  |  | Pos 90 mg bread crust |
| **1766** | F | 2 | AS | 11 |  | 43 | 55 | Pos 13g pasta |
| **134** | M | 8 | AEDS | pos | nd | >100 | nd | Pos 1 g bread |
| **610** | F | 3 | AEDS |  |  |  |  | nd |
| **639** | M | 3 | AEDS, GI |  |  |  |  | nd |
| **642** | F | 5 | AEDS | 9,5 | nd | nd | nd | Pos L.T. |
| **779** | M | 2 | AEDS + Asth | 7 | 8.5 | 93 | 95 | Pos 6 g bread crumb |
| **1026** | M | 12 | AEDS +Asth | 11 | 16 |  |  | Pos Inhalation test |
| **1157** | M | 8 | Urt | 7 | 6 | 29 | 31 | nd |
| **1266** | M | 3.5 | AEDS | 3 | 3 | nd | >100 | nd |
| **1353** | F | 45 | AS | 20 |  | 76.6 | 91 | nd |
| **1494** | M | 13 | AS | 15 | 7 |  |  | nd |
| **1572** |  | 10 |  |  |  |  |  | nd |
| **1638** | M | 13 | AS | 8 | 6 | 73 | 99 | nd |
| **1747** | M | 11 | AS |  |  | 100 |  | nd |
| **1826** | M | 4 | AEDS |  |  | 73 | >100 | nd |
| **1829** | M | 7 | AEDS | pos | pos | >100 | >100 | nd |
| **1830** | M | 3 | AEDS |  |  | 61.4 | 67.1 | nd |
| **1862** |  |  |  |  |  |  |  | nd |
| **1875** |  | 9 |  |  |  |  |  | nd |
